# The Body and Its Ailiments

**Published:** 1876-02

**Authors:** 


THE BODY AND ITS AILMENTS. By George H. Napheys, A.M., M.D. H. C. Watts & Co., No. 1224 Chestnut street, Philadelphia, Pa. Book, pp. 438.

This is a new work, and, as the author expresses it, "Is a hand-book of familiar directions for the treatment of diseases and injuries in both children and adults." It treats of the anatomical arrangement of the body, its organs, etc., and is illustrated by over one hundred engravings and colored plates. Several chapters are devoted to hygiene, and also to the best modes of nursing, etc., in the sick room. The diseases to which the body is heir are explained in a plain, practical way, and the appliances for injuries, such as fractures, dislocations, etc., are simple and easily understood. It is a candidate for popular favor, and will doubtless be so received.

